# Sedation during bronchoscopy: data from a nationwide sedation and monitoring survey

**DOI:** 10.1186/s12890-016-0275-4

**Published:** 2016-08-05

**Authors:** Thomas Gaisl, Daniel J. Bratton, Ludwig T. Heuss, Malcolm Kohler, Christian Schlatzer, Marco P. Zalunardo, Martin Frey, Daniel Franzen

**Affiliations:** 1 Department of Pulmonology, University Hospital Zurich, Rämistrasse 100, 8091 Zurich, Switzerland; 2 Spital Zollikerberg, Zollikerberg, Switzerland; 3 Zurich Centre for Integrative Human Physiology, University of Zurich, Zurich, Switzerland; 4 Centre for Interdisciplinary Sleep Research, University of Zurich, Zurich, Switzerland; 5 Institute of Anaesthesiology, University Hospital Zurich, Zurich, Switzerland; 6 Klinik Barmelweid, Barmelweid, Switzerland

**Keywords:** Bronchoscopy, Propofol, Sedation, Education, Midazolam, Survey

## Abstract

**Background:**

There is limited knowledge on practice patterns in procedural sedation and analgesia (PSA), the use of propofol, and monitoring during flexible bronchoscopy (FB). The purpose of this study was to assess the current practice patterns of FBs and to focus on the use of propofol, the education of the proceduralist, and the involvement of anaesthesiologists during FB.

**Methods:**

An anonymous questionnaire was sent to 299 pulmonologists. Only respondents who were active physicians in adult respiratory medicine performing FB were subsequently analysed.

**Results:**

The response rate was 78 % and 27,149 FB in the previous 12 months were analysed. The overall sedation-related morbidity rate was 0.02 % and mortality was 7/100’000 FB. Sedation was used in 95 % of bronchoscopies. The main drugs used for PSA were propofol (77 %) and midazolam (46 %). In 84 % of PSAs propofol was used without the attendance of an anaesthesiologist. The use of propofol was associated with high volume bronchoscopists (*p* < 0.010) and career-young pulmonologists (*p* < 0.001). While monitoring vital parameters has become standard practice, pulmonologists reported a very low rate of systematic basic education and training in the field of PSA (50 %).

**Conclusions:**

In Switzerland, PSA during FB is mostly performed with propofol without the attendance of an anaesthesiologist and the use of this drug is expected to increase in the future. While monitoring standards are very high there is need for policies to improve education, systematic training, and support for pulmonologists for PSA during FB.

## Background

Flexible bronchoscopy (FB) is still the gold standard for numerous diagnostic and therapeutic interventions and has become an integral part of pulmonary medicine [1]. Sedatives or anaesthetics are a prerequisite for procedural tolerance and patient satisfaction [2–4] and thus they are widely used during the procedure [5]. In general they are considered safe [6] and in the absence of contraindications expert panels explicitly recommend the use of moderate sedation in patients undergoing FB [7–9].

FB is now increasingly performed outside the operating theatre and outside high output tertiary care centres, which has gone hand in hand with a shift in procedural sedation and analgesia (PSA) practice [10]. Historically, anesthesiologists have been predominantly in charge of PSA, but a growing number of proceduralists are managing sedation and anesthesia during FB. This has created debate about bronchoscopist-administered vs. anesthesiologist-administered sedation during bronchoscopy.

Because common complications during FB (e.g. respiratory depression, cardiovascular instability) and one-half of deaths reported during FB are related to sedation, the quality of education, experience and skills of members of the bronchoscopy team are instrumental in maintaining a good safety profile for FB [3]. There is now emerging evidence that proceduralist-administered sedation is feasible [11], safe [6], and cost effective [12]. However, it is unclear to what extent pulmonologists are now in charge of PSA, what their educational background is, and what the current sedation practice entails.

More than 10 years ago, studies suggested the practice of PSA during FB varied greatly for each physician [5, 8, 13]. Despite the wide use of FB among pulmonologists there is little standardisation in practice and the choice of sedative agents [5, 8, 13]. An increasing body of evidence suggests that propofol (2,6-diisopropylphenol) is an eligible anaesthetic agent of choice for PSA during FB [7]. Propofol is a potent hypnotic used for sedation in FB with a fast onset and high degree of controllability [8]. It provokes at least similar sedation, amnesia, and patient tolerance compared with a combination of benzodiazepines and opiates [8]. However, there is no antagonist available and the therapeutic window is narrow. Therefore, the use of non-anaesthesiologist administered propofol (NAAP) remains controversial in several countries due to safety concerns [7, 14]. In Switzerland, propofol is approved for use without the strict legal restrictions applying in some other countries enabling pulmonologists to use it under the umbrella of local guidelines [15]. In contrast to other procedures e.g. gastroenterological endoscopy it is unclear to what extent and under which circumstances propofol is used in PSA during FB [16, 17].

The purpose of this study was: (i) to survey a representative sample of Swiss pulmonologists; (ii) to assess the current practice patterns of FBs; (iii) to focus on the use of propofol and monitoring techniques.

## Methods

### Study population

A structured anonymous and standardised questionnaire was sent to all members of the Swiss Society of Pulmonology (*n* = 299) in their local language using the publicly available database. We excluded participants who did not perform FB in the previous 12 months, did not complete >65 % of questions, or were paediatric pulmonologists. Consequently only physicians (i.e. specialists/consultants) in adult respiratory medicine who recently performed FB in a practice or a hospital were analysed. The survey was conducted from December 2014 until April 2015.

### Questionnaires

The questionnaire was based upon qualitative interviews with experts in this field and earlier studies on PSA [16, 17]. Participants were asked via e-mail to fill out a web-based questionnaire (LamaPoll Berlin, Germany) and state their answers according to their own (internal) documentation. Non-responders were sent a paper-version of the questionnaire. The year of board certification as specialist/consultant was used as a surrogate for length of experience with FB. FB performed by a fellow under direct supervision of a specialist/consultant were not included. The questionnaire addressed the following areas: number of procedures performed during the previous 12 months; sedation rates; frequently used drugs for PSA; personnel involved in PSA and route of drug delivery; use of supplemental oxygen and pharyngeal anaesthesia; frequency and type of monitoring; availability of emergency resuscitative measures; training of personnel involved in PSA; and the incidence of complications attributed to sedation during the previous 12 months. The following questions were conditional on the use of propofol (either regularly or occasionally): usage pattern for propofol (administration or combination scheme); cumulative number of PSA carried out with propofol; and the self-reported number of adverse incidents necessitating emergency intervention during sedation with propofol. In addition, responders were asked to provide detailed qualitative feedback. Replies were anonymous and non-traceable and IP addresses (i.e. computer-numbers) were recorded to identify and delete duplicate records.

### Statistical analysis

Continuous data were summarised as mean ± SD or median (25^th^–75^th^ percentiles) and categorical data summarized using percentages. Continuous variables were compared between groups using the Wilcoxon rank sum test. The association between categorical variables was assessed using Pearson’s χ^2^-test. Risk ratios obtained using binomial regression with a log link function were used to assess the effect of propofol use on various complications adjusting for procedures per year and university hospital (where patients with higher morbidities are more likely to be seen). A two-sided significance level of *p* < 0.05 was considered to be statistically significant. Statistical analysis was performed using STATA version 14 (StataCorp LP, College Station, TX).

### Ethics

Due to the nature of the study, approval from ethics-committee was waived and a declaration of non-objection was issued by the Cantonal Ethics Committee of Zurich, Switzerland (KEK-ZH-Nr. 07-2015). Participants were informed that responding to the questionnaires was their consent to participate.

## Results

### Respondents

**Fig 1:**
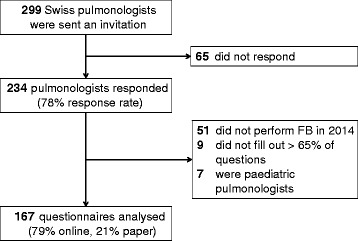
Flow chart of the study. An overall response rate of 78 % was achieved by the use of online questionnaires and reminders (hardcopies on paper) sent via post

**Fig. 2:**
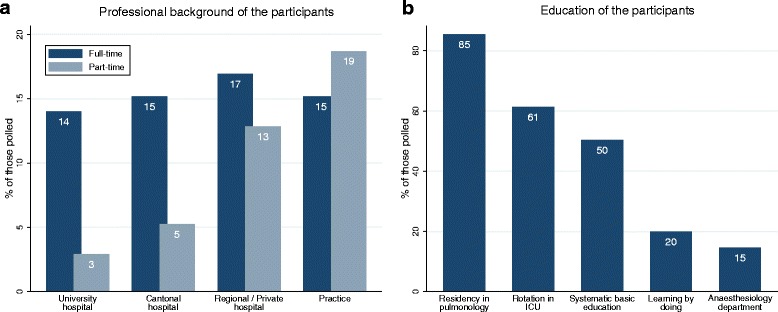
Background and Education. **a** Professional background of the participants. Part-time employment was considered in the analysis. **b** Methods of education & training of Swiss pulmonologists in flexible bronchoscopy. Multiple answers were possible

A total of 299 questionnaires and 83 reminders were sent. The overall response rate was 78 %. After excluding ineligible participants 167 questionnaires were analysed. A detailed summary of the study flow is presented in Fig. 1. The majority (80 %) of the online respondents were German speaking, 18 % were French speaking, and 2 % Italian speaking. In total 27,150 FB from the last year were analysed and respondents performed a median of 60 FB (IQR 30–150) annually. In the year prior to the questionnaire being completed 2 deaths, 290 prolonged apnoeas, and 255 hypotensive events were reported during FB (both defined by a necessary medical intervention). The respondents had on average been board-certified for 14.8 ± 8.8 years. Figure 2 shows the professional background and education of the participants. The overall FB-related mortality rate was 7/100’000 FB. The sedation-related overall morbidity rate (defined as needing a medical intervention needed) was 0.02 %. The most common complications during FB were apnoeas (defined as the need for bag-ventilation) with a rate of 0.011 % and hypotonia (defined as the need for intervention) with a rate of 0.009 %.

### Sedation

**Fig 3:**
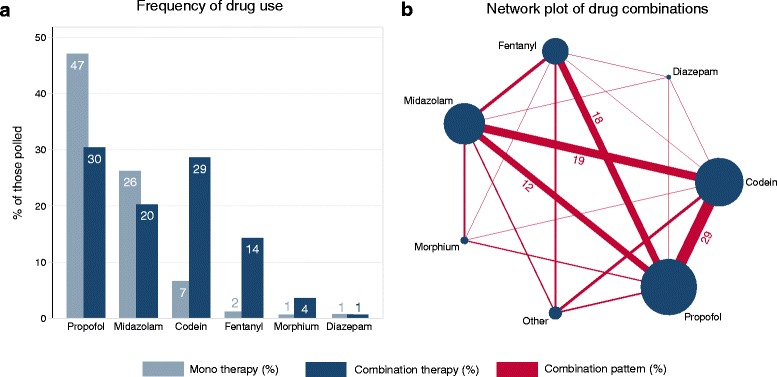
Sedatives **a** Frequency of sedatives/hypnotics used for flexible bronchoscopies by survey participants. Sedatives/hypnotics in general were used in a median of 100 % (IQR 90–100) of flexible bronchoscopies. 77 % of respondents reported the use of propofol on a regular basis (either mono or combination therapy). The most common mono therapy was propofol (47 %) and the most common combination therapy was propofol + codein (11 %, data not shown). Combination therapy included the combination of two or more drugs. **b** Network plot of drug combinations. The relative size of the circles/bars represents the frequency of the usage/combination. Combination patterns ≤5 % are not labelled. Combination patterns are shown in % of all possible combinations. IQR = Interquartile range

Pulmonologists reported using sedatives or hypnotics in a median of 100 % (IQR 90–100) of FBs. Propofol was by far the most popular regularly used drug among Swiss pulmonologists (77 %) either as a mono use (47 % of the respondents) or in combination with other sedatives (30 % of the respondents). A detailed distribution of the frequency of drug use and combinations is shown in Fig. 3. Proceduralists were mostly assisted by two staff members (58 %) or one (41 %) during FB (1 % reported more than 2 staff members).

### Administration of drugs

The drugs were mostly administered by a nurse supervised by the bronchoscopist (73 %) less frequently by an anaesthesiologist (14 %), the proceduralist him/her-self (10 %), or other personnel (3 %). Most of the physicians administered the drug via a peripheral venous catheter either with sodium chloride (65 %) or without (12 %). Other administration routes included: 13 % perfusor, 3 % direct intra-venous, 3 % intra-muscular, and 5 % other administrations routes. In the case of propofol the drug was administered in 70 % as a bolus and in 30 % with the help of a perfusor.

### Monitoring

The use of electronic monitoring by means of blood pressure (87 %), pulse (95 %), and oxygen saturation (98 %) is currently standard practice in Switzerland and was routinely applied by the majority of respondents. In patients with multiple comorbidities (e.g. patients with lung diseases; obese patients; patients with obstructive sleep apnoea) the monitoring rate was almost 100 %. ECG (41 %) and capnography (10 %) was only applied in selected patients. New emerging technologies like non-invasive ventilation (3 %) and transcutaneous CO2-monitoring (1 %) were only applied in specialised centres. The on-site availability of emergency measures was: additional O2-tank (96 %), flumazenil (85 %), naloxone (77 %), atropine (83 %), oropharyngeal airway (72 %), bag-valve-mask ventilation (96 %), tracheal intubation (78 %), defibrillator (84 %), and resuscitation team (85 %).

### Propofol

**Fig. 4:**
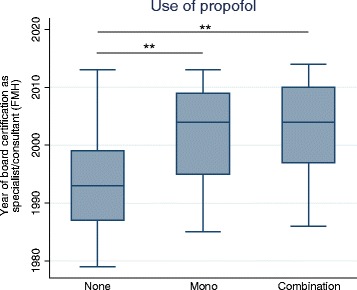
Propofol and career-age. Box plot summarising the career-age of pulmonologists either not using propofol or using it alone or in combination. Career-young pulmonologists were more likely to use propofol (either mono or in combination) for procedural sedation and analgesia of their flexible bronchoscopy-patients. The career-age was determined by the year of board certification as specialist/consultant (by the Swiss Medical Association [FMH]). ** *p* < 0.001

Propofol was the most commonly administered drug during FB, without a significant preference of a certain hospital type (Table 1). Anaesthesiologists used propofol in 57 % of cases and were more likely to use propofol when compared to non-anaesthesiologists (*p* = 0.023). For 84 % of respondents propofol was used without the attendance of an anaesthesiologist. Respondents who used propofol in the last year reported using propofol for a median of 5 years (IQR 2–6.5 years) and have performed a median of 200 sedations (IQR 7.5–500) with the drug in their career. During the past 12 months approx. 21,140 procedures were carried out by respiratory physicians using propofol without the assistance of an anaesthesiologist. Physicians who used propofol (either alone or in combination with another drug) performed more FB procedures per year when compared to physicians who did not use propofol at all (100 [IQR40–180] vs 45 [IQR 20–100]; *p* < 0.010) and were also at an earlier stage of their career (Fig. 4). The use of propofol could not be attributed to a specific language region (*p* = 0.480) or discharge time (*p* = 0.695). When adjusted for procedures per year (log transformed) and university hospitals, the use of propofol was not associated with reported apnoeas (RR = 1.32; 95 % CI 0.89–1.96; *p* = 0.17) or events of hypotension (RR = 1.07; 95 % CI 0.69–1.67; *p* = 0.77) during the procedure.

**Table 1:** Summary statistics of propofol use by different hospital types

	University hospital (*n* = 22)	Cantonal hospital (*n* = 30)	Regional/Private hospital (*n* = 23)	Practice (*n* = 21)
Propofol use, %	85 %	91 %	71 %	65 %
Bolus/Perfusor, %	68 % / 32 %	68 % / 32 %	92 % / 8 %	52 % / 48 %
Attendance of anaesthesiologists, %	9 %	3 %	0 %	43 %
Median number of procedures/year (IQR)	100 (50–450)	170 (130–300)	100 (40–150)	27 (15–50)

For this analysis, only respondents who were affiliated with only one hospital type were analysed (*n* = 96). While most of flexible bronchoscopies were performed in cantonal hospitals anaesthesiologists mostly attended flexible bronchoscopies in a practice. IQR = Interquartile range

## Discussion

The administration of sedation during FB by the bronchoscopist is controversial with large variations in different countries. This survey assesses the current practice patterns among most Swiss pulmonologists and gives insights into the widespread use of propofol during FB in Switzerland. It demonstrates a high heterogeneity in PSA practice patterns which is influenced by local preferences and was also observed in other studies [5, 13]. In contrast to earlier studies in this field, the current study is distinct with regard to (i) the relatively high sedation rate; (ii) the widespread use of propofol even in private practice without the attendance of an anaesthesiologist; (iii) and a high monitoring level [5, 13]. We hypothesized that these findings can be attributed to a shift in practice as well as country-specific preferences. However, the results may also have important clinical implications with regard to future education and bronchoscopists in other countries who would like to change their PSA patterns (e.g. towards the usage of propofol).

### Safety results

The reported mortality and complication rate during FB is generally low but comparable to other studies in this field [18, 19]. The data suggests that FB is generally a safe procedure associated with only few complications and, surprisingly, the reported incidence of apnoeas in our study was lower compared to gastro-intestinal endoscopies [17, 20]. A possible explanation for the diverging complication rates to other specialities lies in the fact that pulmonologists tend to have more knowledge about airway management and breathing problems. Perhaps, this could also be due to the fact that (compared to Swiss gastroenterologists) the pulmonologists were faced for the first time with a survey of this kind.

### The use of propofol

The results confirm a trend towards propofol as an agent of choice. In 1989 propofol was approved in Switzerland and initially used around 2000 by non-anaesthesiologists for gastro-intestinal endoscopy and FB [15, 20]. Propofol has emerged as an appealing agent of choice: it is better tolerated by patients with a faster recovery time and a quicker return to baseline mental status [21–24]. The novelty of the drug and individual familiarity with pre-existing regimens are also most likely to be the reasons for a lower proportion of late-career pulmonologists using propofol. Thus, the use of propofol is predicted to rise in the future (Fig. 4). It is important to keep in mind that in Switzerland, propofol is approved for use without the strict legal limitations that exist in some other countries. In particular, non-anaesthesiologists are allowed to use propofol provided they have had an adequate training for its use. For example at the University Hospital Zurich in agreement with the Institute of Anaesthesiology the following recommendations are given: First, the proceduralist should not be the same person who is administering the drug. Second, the patient should be adequately monitored (at least oxygen saturation and blood pressure). Lastly, the proceduralist and the drug administering person should be capable of airway management and emergency ventilation. The rate of proceduralist-administered propofol sedation of 84 % was also very high and, to our knowledge, the highest reported rate so far. However, worldwide the use of NAAP remains controversial since propofol lacks an available antagonist and its narrow therapeutic window between no sedation and deep sedation with apnoea warrants a formally trained physician. Gastroenterologists in Switzerland have shown that NAAP is safe, especially for gastro-intestinal endoscopy [25]. In addition, other studies have shown that nurse-administered propofol is a feasible and safe sedative during FB [26]. Nevertheless, the cooperation between anaesthesiologists and bronchoscopists remains crucial [10]. Of those physicians who used propofol, 43 % reported that they were actively supported or trained by an anaesthesiologist at the time of the introduction of the drug. This is a very similar rate to Swiss gastroenterologists performing endoscopy [17]. In general, our data suggest that propofol administration represents a safe sedation technique that can be performed by a non-anaesthesiologist [23]. This aspect is particularly relevant since the advantages of propofol e.g. fast onset and high degree of controllability makes it a drug of choice for PSA during FB [8].

### Clinical implications

The study has some significant clinical implications. First, the data gives insight into a health-care system where the majority of pulmonologists are using propofol for PSA during FB. It shows that NAAP is feasible and can work on a large scale without an increased rate of serious adverse events. Second, the study raises awareness that these practice patterns clearly result in augmented educational demands for pulmonologists (specifically, training in NAAP) which should be taken into account. It is vital that a bronchoscopist can facilitate every aspect of PSA during FB, especially adequate and efficient therapy of complications.

The overwhelming part of qualitative feedback provided in this study concerned the education of pulmonologists in PSA and uncertainties regarding the competences of proceduralists. Only 50 % of the respondents stated that “systematic basic education” in the field of PSA was part of their education. This is in line with other countries (e.g. Italy [27]) and provides evidence for professional societies and certifying agencies to move from a volume-based certification system (which often implies “learning by doing” [Fig. 2b]) to a standardised skill acquisition and knowledge-based competency assessment, as already recommended by expert panels [28]. Currently, the European guidelines state minimum numbers of supervised procedures required for a trainee to be deemed competent [29]. A meta-analysis has shown that systematic FB-training programs (ideally before exposure to patients) are effective in terms of skills and behaviours in comparison with no intervention and they can also be used to harmonise PSA in the field of FB [30]. The skillset of future interventional pulmonologists comprises the independent performance of PSA during FB. This skill should not depend on potential rotations e.g. former rotations in the intensive care unit but rather should be systematically trained [27].

### Limitations and future studies

A limitation of this study is that the data are self-reported and do not originate from registers. However, by anonymising the questionnaire we sought to minimise the underestimation of “unpleasant” data and encourage participants to accurately state their complication numbers. On the other hand, because of the anonymity, no further data beyond our questionnaire (e.g. circumstances of the two reported deaths, FB indications, patient data etc.) could be retrieved. The fact that the response rate of this study (78 %) is higher than comparable studies [20] and that answers were retrieved from all 15 major hospitals (five university hospitals and ten cantonal hospitals) in Switzerland the authors feel confident that the presented data are reliable and represent current clinical practice. Additionally, the observed mortality rates are comparable to those from other countries, a fact which promotes confidence in the report of yet “unpleasant data” [18, 19]. Under the assumption that sedation practice does not differ among pulmonologists in each centre the “unknown” sedation practice of the 22 % non-responders is likely to have a negligible effect on the presented data. Finally, the study represents only cross-sectional PSA practice patterns. Experience in gastroenterological studies has also taught us that sedation practice is constantly evolving and that the picture of current sedation patterns can change drastically within a relatively short time period [16, 17]. Although we have tried to anticipate the future usage patterns of propofol (Fig. 4), there is a need for further evaluations (including electronic databases) in this field to accurately assess shifts in PSA practice patterns. Since the data is representative for Switzerland only, further surveys in other countries are needed.

## Conclusions

This representative survey of Swiss pulmonologists demonstrates that the majority of Swiss pulmonologists are in charge of PSA of their patients and describes in detail the current sedation and monitoring practice. Propofol was by far the most commonly administered drug during FB in adults and the dominance in PSA of this drug is likely to increase in the future. This study highlights the need of education and systematic training of pulmonologists for PSA during FB.

## Abbreviations

FB, flexible bronchoscopies; IQR, interquartile range; NAAP, non-anaesthesiologist administered propofol; PSA, procedural sedation and analgesia; SD, standard deviation
